# The behavioral, cellular and immune mediators of HIV-1 acquisition: New insights from population genetics

**DOI:** 10.1038/s41598-020-59256-0

**Published:** 2020-02-24

**Authors:** Timothy R. Powell, Rodrigo R. R. Duarte, Matthew Hotopf, Stephani L. Hatch, Miguel de Mulder Rougvie, Gerome D. Breen, Cathryn M. Lewis, Douglas F. Nixon

**Affiliations:** 1 Social, Genetic & Developmental Psychiatry Centre, Institute of Psychiatry, Psychology & Neuroscience, King’s College London, London, UK; 2 Division of Infectious Diseases, Weill Cornell Medicine, Cornell University, New York, NY USA; 3 Department of Psychological Medicine, Institute of Psychiatry, Psychology & Neuroscience, King’s College London, London, UK

**Keywords:** Genetic variation, Infectious diseases

## Abstract

Millions are exposed to the human immunodeficiency virus type 1 (HIV-1) every year, but not all acquire the virus, suggesting a potential role for host genetics in the moderation of HIV-1 acquisition. Here, we analyzed summary statistics from the largest genome-wide association study of HIV-1 acquisition to-date, consisting of 6,334 infected patients and 7,247 population controls, to advance our understanding of the genetic mechanisms implicated in this trait. We found that HIV-1 acquisition is polygenic and heritable, with SNP heritability estimates explaining 28–42% of the variance in this trait at a population level. Genetic correlations alongside UK Biobank data revealed associations with smoking, prospective memory and socioeconomic traits. Gene-level enrichment analysis identified EF-hand calcium binding domain 14 as a novel susceptibility gene for HIV–1 acquisition. We also observed that susceptibility variants for HIV-1 acquisition were significantly enriched for genes expressed in T-cells, but also in striatal and hippocampal neurons. Finally, we tested how polygenic risk scores for HIV-1 acquisition influence blood levels of 35 inflammatory markers in 406 HIV-1-negative individuals. We found that higher genetic risk for HIV-1 acquisition was associated with lower levels of C-C motif chemokine ligand 17. Our findings corroborate a complex model for HIV-1 acquisition, whereby susceptibility is partly heritable and moderated by specific behavioral, cellular and immunological parameters.

## Introduction

Around 38 million people currently live with the human immunodeficiency virus type 1 (HIV-1) worldwide^1^, and millions more are exposed to potential infection every year through sexual contact, vertical transmission, or via the parenteral route^2,3^. First-line prevention strategies against acquisition comprise of the use of condoms and pre-exposure prophylaxis (PrEP), or abstinence from drugs or sex^4^. However, epidemiological studies have identified varying degrees of susceptibility to HIV-1, suggesting that host genetics may play a role in moderating acquisition, which could be explored in the context of preventive strategies. For example, studies conducted prior to the development of antiretroviral therapy observed that less than a third of babies born from HIV-1-positive mothers acquire HIV-1^5^ and, similarly, that a proportion of highly exposed individuals are resistant to infection^6^. Supporting this hypothesis, homozygosity of the Δ32 mutation of the C-C chemokine receptor type 5 (*CCR5*) gene has been shown to protect against HIV-1 infection^7–9^, as the encoded protein is a co-receptor needed for viral entry. However, it remains unknown whether common genetic risk factors are also involved in host susceptibility to acquisition.

HIV-1 acquisition is a complex phenotype that consists of behavioral risk parameters and biological factors moderating viral entry and replication. A better understanding of both behavioral and biological factors influencing acquisition has the potential to improve our basic comprehension of acquisition, inform prevention strategies and clinical trials, and reduce social stigma. In this context, genome-wide association studies (GWAS) provide a powerful means to identify variants and risk mechanisms implicated in HIV-1 acquisition. No genome-wide significant polymorphisms have been robustly associated with HIV-1 acquisition to-date^10–16^. However, population genetic methods have developed substantially in recent years, now allowing for powerful, biologically-informative analyses even in moderately-sized GWAS. For example, gene enrichment analyses can be applied to summary statistics to identify genes involved in risk, as well as cell types more likely mediating susceptibility. This is achieved by averaging association signals from multiple neighboring polymorphisms within protein-coding genes, and comparing lists of the resulting genes to the transcriptional profiles of mammalian cell types^17,18^. Methods like Linkage Disequilibrium Score Regression (LDSC)^19^ and Linkage Disequilibrium Adjusted Kinships (LDAK)^20^ further allow the estimation of SNP heritability based on GWAS results. Polygenic risk scores (PRS), in turn, can be used to explore genetic overlap between traits, and are useful in establishing the effect genetic predisposition has on biological parameters. PRS could even be used to model genetic risk for a trait (e.g. HIV-1 acquisition susceptibility) in a cohort of unaffected individuals (e.g. HIV-1-negative individuals). This, for example, could allow us to model the impact of genetic predisposition to HIV-1 acquisition on biological systems prior to HIV-1 infection, which could ultimately aid in the identification of new vaccine or drug development strategies. Here, we apply these modern population genetic methods to the largest GWAS of HIV-1 acquisition, and identify novel factors and complex traits associated with HIV-1 acquisition.

## Results

### HIV-1 acquisition is heritable and correlates with behavioral and socioeconomic traits

The largest GWAS meta-analysis of HIV-1 acquisition to-date tested over 8 million common polymorphisms for association with HIV-1 acquisition in 6,334 infected patients and 7,247 population controls^10^. We estimated the SNP heritability (SNP h^2^) of HIV-1 acquisition by analyzing these GWAS results using LD Hub (LDSC-based)^19^ and SumHer-GC^20^ (based on the LDAK model, correcting for possible hidden population structure). We observed that SNP h^2^ = 0.28 ± 0.05 (standard deviation; SD) under the LDSC model, suggesting that HIV-1 acquisition is a heritable trait, replicating a previous analysis of the same dataset^21^. Analysis with SumHer-GC showed a higher estimate of SNP h^2^ (0.42 ± 0.08), consistent with LDAK being able to capture a larger proportion of SNPs contributing to SNP h^2^, relative to LDSC.

**Figure 1:**
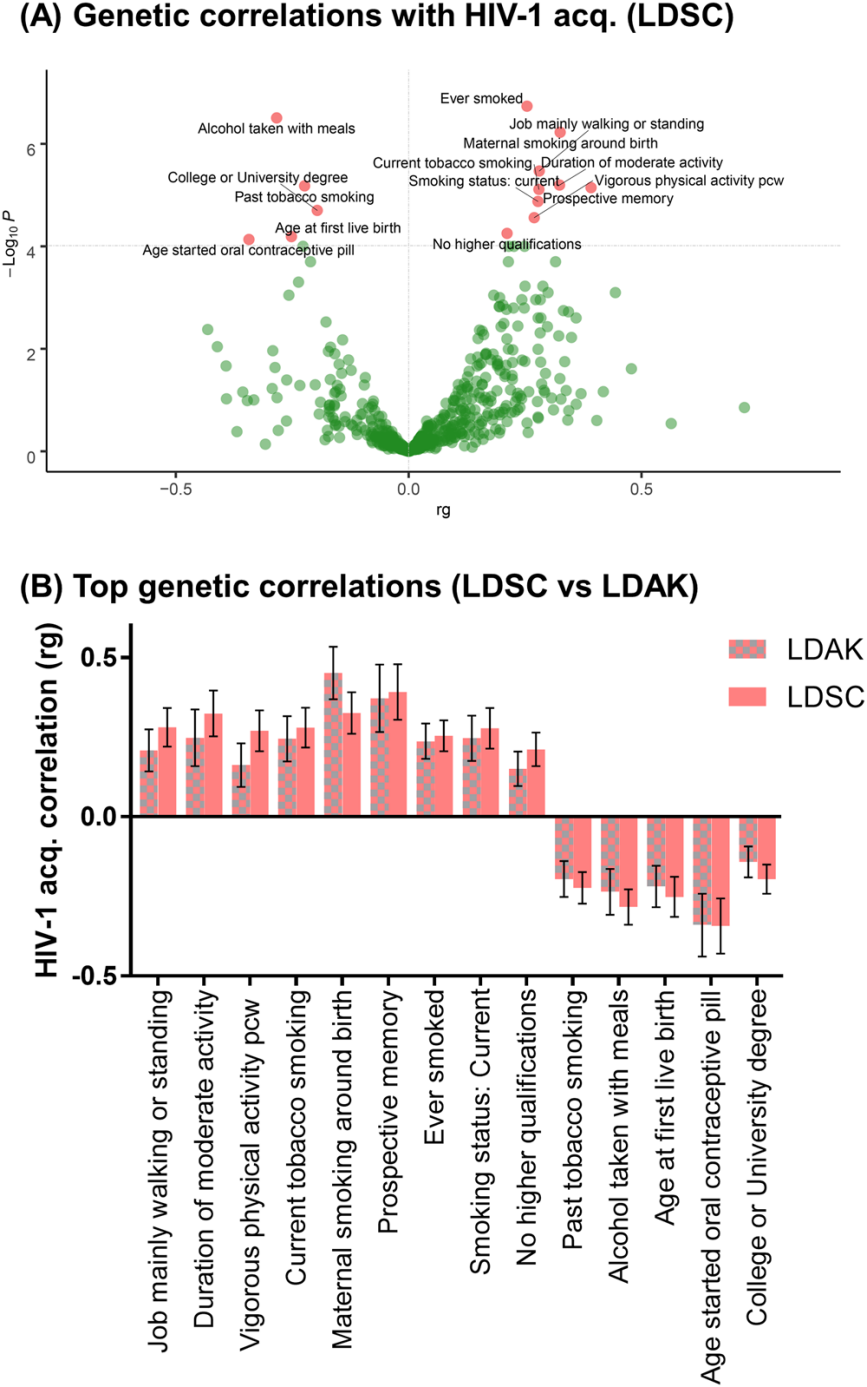
Genetic correlations between HIV-1 acquisition susceptibility and traits tested within the UK Biobank. **(A)** Correlations performed within LD Hub using the LDSC model to determine genetic correlations between HIV-1 acquisition and 516 heritable traits. The Bonferroni-significant correlations (rg) are displayed in red and delimited by the horizontal line (P = 0.05/516 traits = 8.38 × 10^−5^). **(B)** Validation of the Bonferroni-significant findings using SumHer-GC, which is based on the LDAK model, adjusting for genomic control. The correlation values observed for the 14 Bonferroni-significant traits associated with HIV-1 acquisition in the LDSC method were highly concordant with results using the LDAK method (Pearson’s r = 0.98, P = 2.59 × 10^−9^).

To better understand which behavioral parameters might be important in moderating HIV-1 acquisition, we performed genetic correlation analyses leveraging on GWAS data from 516 heritable traits assessed in the UK Biobank via LD Hub, which contains genetic association results from up to 488,377 individuals. This LDSC-based analysis revealed 9 positive correlations with HIV-1 acquisition, including *prospective memory,* ascertained using cognitive tests (rg = 0.39, SE = 0.09, P = 7.15 × 10^−6^); lower levels of education, ascertained by having *no higher qualifications* (rg = 0.21, SE = 0.05, P = 5.59 × 10^−5^); self-reported *smoking* (rg = 0.28, SE = 0.06, P = 1.33 × 10^−5^); and self-reported *vigorous exercising* (rg = 0.27, SE = 0.06, P = 2.76 × 10^−5^; Bonferroni adjusted P (for 516 traits) <0.05 for all; Fig. 1A; Supplemental Table 1). We also observed 5 negative associations with acquisition, including socioeconomic traits like *alcohol intake with meals* (rg = −0.28, SE = 0.06, P = 3.12 × 10^−7^), *having a higher qualification* (rg = −0.20, SE = 0.05, P = 1.99 × 10^−5^), and *age at which female participants had their first live birth* (rg = −0.25, SE = 0.06, P = 6.46 × 10^−5^). We validated the Bonferroni-significant genetic correlations using SumHer-GC (Fig. 1B), and observed highly concordant estimates of genetic correlation calculated between the two methods (Pearson’s r = 0.98, P = 2.59 × 10^−9^).

### *EFCAB14* as a novel susceptibility gene for HIV-1 acquisition

**Figure 2:**
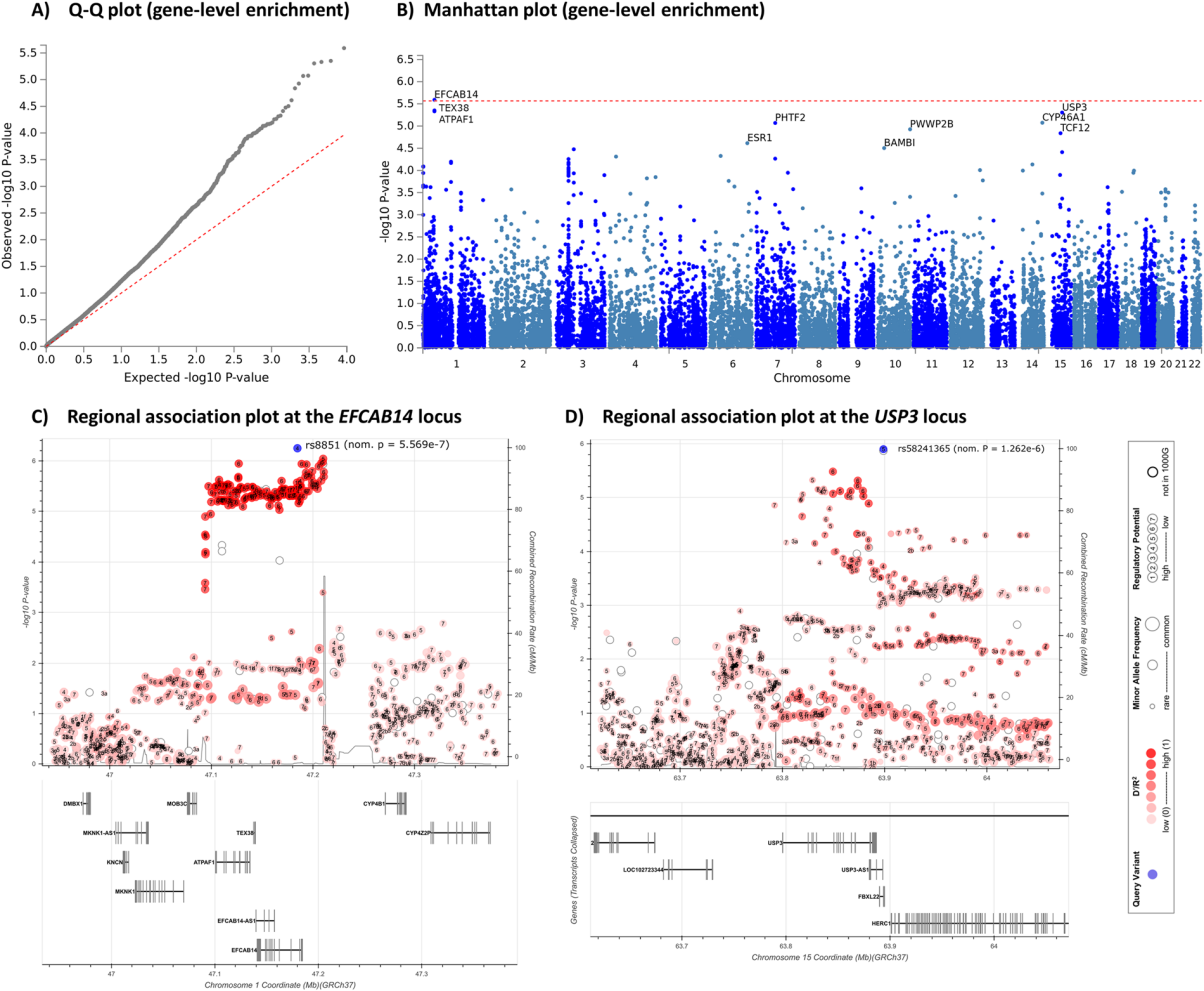
Gene-level enrichment analysis of the HIV-1 acquisition GWAS summary statistics identified a Bonferroni-significant susceptibility gene. (**A)** The quantile-quantile (Q-Q) plot shows the high number of observed genes associated with HIV-1 acquisition, compared to the number of expected genes assuming a normal distribution (dotted red line). Generated using FUMA^17^. (**B)** Gene-level Manhattan plot showing the novel susceptibility gene for HIV-1 acquisition on chromosome 1p33, *EFCAB14*. Generated using FUMA^17^. (**C)** Regional association plot demonstrating high linkage disequilibrium at the *EFCAB14* locus. Generated using LDassoc^51^. (**D)** Regional association plot at the second highest association signal with HIV-1 acquisition outside of chromosome 1. Generated using LDassoc^51^.

To perform our gene-level enrichment analysis, gene-level statistics and weighted p-values were generated from GWAS summary statistics using MAGMA^22^, adjusting associations for gene size, single nucleotide polymorphism (SNP) density and linkage disequilibrium. This analysis revealed a contribution of several protein-coding genes to HIV-1 acquisition, with gene-level Q-Q plots highlighting the degree of polygenicity observed, and the abundance of contributing genes relative to an expected normal distribution (Fig. 2A; Supplemental Table 2). We identified a novel gene involved in HIV-1 acquisition, the EF-hand calcium binding domain 14 (*EFCAB14*), on chromosome 1p33 (Z-score = 4.56, enrichment P = 2.56 × 10^−6^, Bonferroni corrected P = 4.7 × 10^−2^), which is expressed ubiquitously across tissues, according to The Genotype-Tissue Expression (GTEx) project^23^. The highest association signal at this locus is the non-coding variant rs8851 (P = 5.57 × 10^−7^; Fig. 2B), which is also an expression quantitative trait loci (eQTL) for *EFCAB14*. The risk (G-) allele of rs8851 is associated with lower expression of *EFCAB14* in multiple tissues, including whole blood, skin, adipose tissue, the cerebellum and arteries (P < 1 × 10^−3^ for all)^23^. These findings suggest that genetic risk for HIV-1 acquisition at this locus is conferred via reduced expression of *EFCAB14*, and not by altered expression of neighboring genes such as the ATP synthase mitochondrial F1 complex assembly factor 1 (*ATPAF1*) or the testis expressed 38 (*TEX38*) genes (Fig. 2C). Outside of chromosome 1p33, the top gene-level association signal was on chromosome 15q22.31, at the ubiquitin specific peptidase 3 gene (*USP3*), although this was not significant after multiple testing correction (enrichment P = 4.96 × 10^−6^, Bonferroni corrected P > 0.05; Fig. 2D).

### HIV-1 acquisition susceptibility variants are enriched within genes expressed in T-cells and striatal neurons

**Figure 3:**
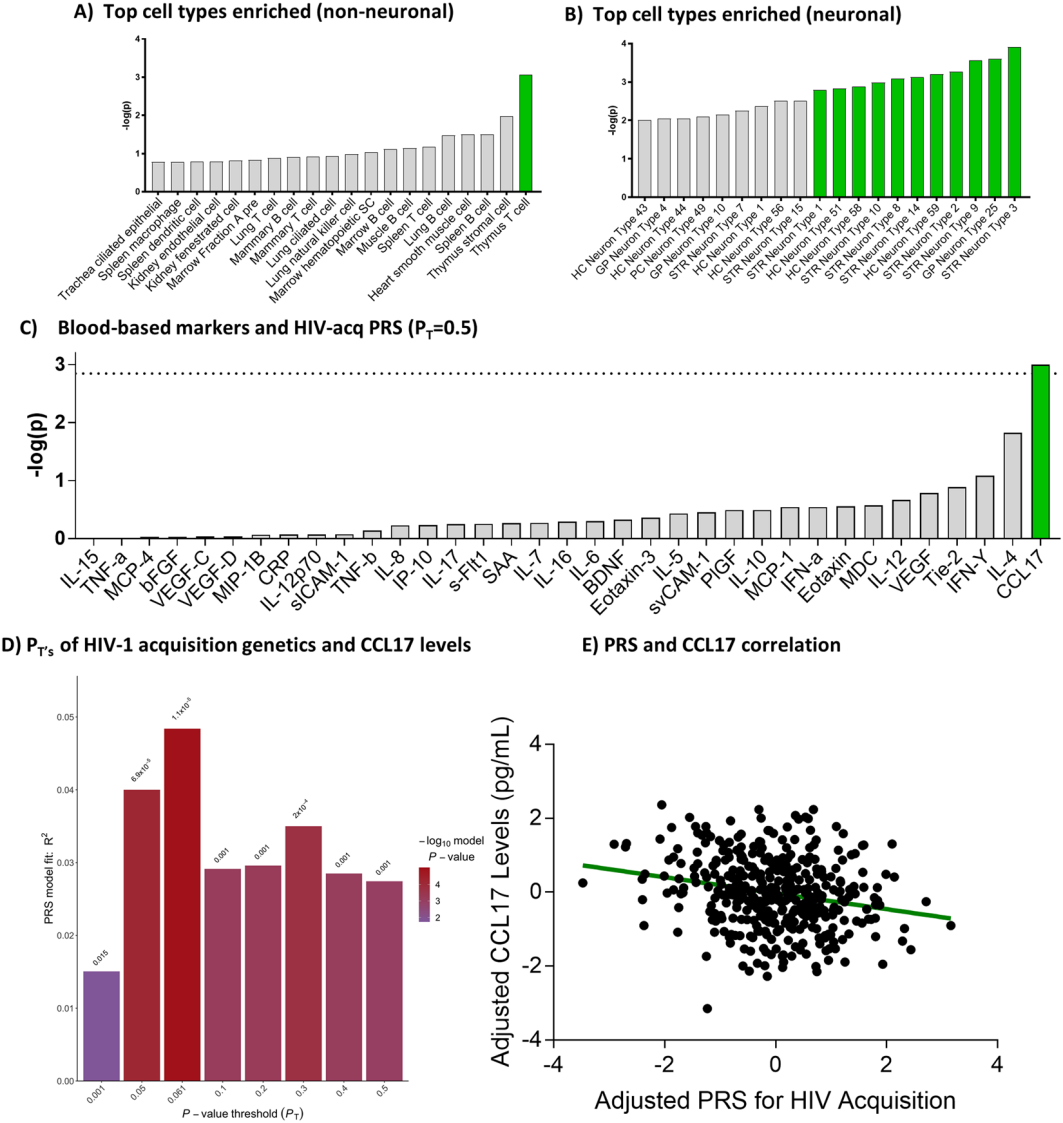
HIV-1 acquisition genetics significantly overlaps with the expression profile of T-cells and neurons, and correlates with blood levels of CCL17. (**A)** Top 20 enrichment signals observed for murine non-neuronal cell types. (**B)** Top 20 enrichment signals observed for murine neuronal cell types. Green indicates significance under a false discovery rate of 10%. (**C)** Plot showing -log(p) of the association between 35 markers and a polygenic risk score for HIV-1 acquisition. The dashed line indicates Bonferroni significance. **(D)** Sensitivity analysis revealed that PRS at every P_T_ significantly correlated with CCL17 levels in blood, with the highest significance at P_T_ = 0.061. (**E)** Correlation between PRS for HIV-1 acquisition (P_T_ = 0.061) adjusted for seven population dimensions, and CCL17 levels adjusted for age, gender, BMI, ethnicity, and smoking status.

We aimed to investigate the cellular basis for the biological and behavioral parameters implicated in HIV-1 acquisition, and therefore investigated the cell types enriched for variants associated with this trait. Two independent gene-set enrichment analyses using FUMA^24^ showed that cells likely mediating acquisition included T-cells (P = 8.54 × 10^−4^) and, independently, neurons from the striatum, hippocampus and globus pallidus under a false discovery rate of 10% (top association signals, respectively: P = 1.22 × 10^−4^, 2.49 × 10^−4^, and 6.23 × 10^−4^; q < 0.10 for all; Fig. 3A,B, Supplemental Tables 3 and 4).

### Polygenic risk score for HIV-1 acquisition is negatively associated with circulating CCL17 levels

We utilized findings from the GWAS performed by McLaren and colleagues (2013) to calculate PRS for HIV-1 acquisition in an unrelated cohort of HIV-1-negative individuals, to measure how acquisition predisposition correlated with levels of 35 blood-based inflammatory markers. Our rationale was to better understand how genetic susceptibility expresses itself in the pre-exposed immune system, and in particular how it affects inflammatory cytokines, which are molecules that can be modified via pharmacological intervention, and are thought to be key moderators of HIV-1 infection^25,26^. A preliminary analysis utilized all polymorphisms associated with HIV-1 acquisition under a P association threshold (P_T_) = 0.5 to calculate the PRS, and to test its correlation with the levels of 35 inflammatory markers. This analysis showed that HIV-1 acquisition was significantly and specifically associated with lower levels of the chemokine CCL17 (ß = −1644.32, standard error (SE) = 496.62, P = 1.00 × 10^−3^, Bonferroni corrected P (for 35 tests) = 3.50 × 10^−2^, variance explained = 2.74%; Fig. 3C). A sensitivity analysis revealed that PRS at every tested P_T_ also significantly predicted levels of this chemokine, with highest significance at P_T_ = 0.061 (ß = −800.52, SE = 180.07, P = 1.14 × 10^−5^, corrected P = 5.11 × 10^−4^, variance explained = 4.84%; Fig. 3D). Importantly, this effect was additionally observed when considering individuals of European-only ancestry in the cohort (ß = −794.27, SE = 190.81 P = 4.21 × 10^−5^), matching the ethnicity of the individuals in the base GWAS used to construct the PRS, and after removing the major histocompatibility complex from the PRS calculation (ß = −526.42, SE = 158.08, P = 9.50 × 10^−4^). The inverse correlation observed suggests that individuals that have a higher genetic predisposition to HIV-1 acquisition are more likely to have lower blood levels of CCL17 (Fig. 3E).

## Discussion

HIV-1 acquisition consists of behavioral risk parameters moderating exposure as well as biological factors controlling viral entry and replication. However, the genetic aspects of HIV-1 acquisition (outside of the CCR5 Δ32 mutation) have been understudied, in part because no robust genome-wide significant variants were found in early studies^10–16^, which led to the premature assumption that acquisition was not substantially moderated by common genetic variants. The GWAS analyzed here, by McLaren and colleagues (2013), did not identify genome-wide significant polymorphisms associated with HIV-1 acquisition (after correcting association signals for frailty bias), but population genetic methods have advanced considerably in recent years, now allowing for powerful inferences about genetic traits using GWAS summary statistics^19,22,24,27^, even in moderately powered studies. For instance, we calculated heritability estimates of HIV-1 acquisition using cutting edge methods like LDSC and LDAK, which would otherwise be challenging using traditional twin and family methods. The level of SNP h^2^ observed for acquisition (LDSC: 0.28 ± 0.05; LDAK: 0.42 ± 0.08) was greater or comparable to that of traits considered highly heritable, such as body mass index (LDSC: 0.09 ± 0.01; LDAK: 0.33 ± 0.03), height (LDSC: 0.20 ± 0.02; LDAK: 0.46 ± 0.04), and schizophrenia (LDSC: 0.19 ± 0.01; LDAK: 0.42 ± 0.02)^20^. Overall, these results highlight the contribution of common variants to HIV-1 acquisition risk, showing this is a heritable trait.

To understand the underlying genetic factors associated with HIV-1 acquisition, we performed genetic correlation analyses in LD Hub, which leverages on data from ~500,000 individuals from the UK Biobank, to investigate how acquisition genetics correlates with heritable traits assessed in this large population sample. We observed genetic correlations between HIV-1 acquisition and heritable phenotypes associated with socio-economic factors, corroborating previous epidemiological work, and further highlighting the need for prevention strategies tailored to individuals who most need it^28^. We further validated the genetic correlations using the independent SumHer-GC method, supporting these results.

Our results also validate and expand the current understanding of the biological basis of HIV-1 acquisition. In a preliminary enrichment analysis that aimed to identify the cell types that mediate HIV-1 acquisition susceptibility throughout the body, we observed that polymorphisms implicated in acquisition were enriched for genes expressed in T-cells, which are the main targets for HIV-1 replication^29^. We further tested the enrichment of HIV-1 variants for genes expressed across a range of neural cell types, since these cells mediate behavior and could explain certain genetic correlations observed with HIV-1 acquisition. We observed a significant enrichment for striatal and hippocampal neurons in association with HIV-1 acquisition, which is particularly striking considering they are brain areas implicated in the regulation of reward and pleasure^30,31^. Alternatively, these cell types may represent those which harbor HIV-1 and most effectively hide it from the immune system, propagating a sustained infection. Furthermore, the gene-level enrichment analysis identified *EFCAB14* as a susceptibility gene for HIV-1 acquisition, on chromosome 1p33. This gene is ubiquitously expressed in the body, and the risk allele of rs8851 is known to reduce expression of *EFCAB14* across multiple tissues. Proteins containing EF-Hand Calcium Binding domains in general are implicated in functions ranging from intracellular calcium buffering, signal transduction and muscle contraction^32^, but future studies are warranted to investigate the function of *EFCAB14* specifically, particularly in the context of HIV-1 acquisition.

Another emerging method in population genetics is polygenic risk scoring^33,34^. We modelled how genetic risk for HIV-1 acquisition expresses itself in the pre-exposed immune profile using a cohort of HIV-1 negative individuals. By considering genetic risk as a continuous trait in a population setting, we can more powerfully determine the influences of the genetic risk signal on innate biological systems such as inflammatory marker expression, without confounders (e.g. drug use, other infections) more commonly associated with individuals from high-risk groups. Moreover, previous studies that have compared high-risk individuals who do not acquire HIV-1, with those that do, are likely confounded by the fact that HIV-1 has an influence on the immune system and inflammatory profile of the individual, which may not correspond to the pre-exposed immune profile associated with risk or resilience. In particular, we studied how genetic predisposition to HIV-1 acquisition affects inflammatory cytokines, which are immune messengers that are relatively easy to assay, can be modified via pharmacological intervention, and are thought to be key moderators of HIV-1 infection^25,26^. We observed that PRS for HIV-1 acquisition inversely correlated with CCL17 levels in the blood of HIV-1 negative individuals, suggesting that levels of this chemokine should be considered in clinical trials for biomarker, drug and vaccine development. CCL17 is known to regulate the development and maturation of T-cells in the thymus, as well as their trafficking during inflammation^35–37^. Neutralization of this chemokine by antibody treatment has been shown to block the recruitment of T-cells in the lung (ameliorating respiratory allergy)^38^. We hypothesize that increased CCL17 levels may increase the influx of inflammatory cells, which could help eliminate HIV-1-infected cells before the establishment of a systemic infection. However, CCL17 levels may represent only one of many biological mechanisms implicated in, or co-occurring with, HIV-1 acquisition susceptibility, and further research is needed to better understand this relationship.

Despite the insights provided, our study has limitations, including the modest cohort size of European-only individuals in the GWAS analyzed. Analyses of larger cohorts from different ancestry groups and well-characterized infection routes have the potential to improve our understanding of HIV-1 acquisition, by improving the identification of specific SNPs and genes involved. Future well-powered GWAS of HIV-1 acquisition comparing high risk individuals who are infected versus those who are not will more likely tease apart the biological risk mechanisms implicated in viral resilience from the behavioral risk factors. This could be studied in areas where the risk for HIV-1 is already relatively high in the general population (e.g. South Africa), and where viral entry and replication may represent more important components of acquisition than risk behaviors. Furthermore, although we validated our estimates of heritability and genetic correlations by using two independent methods, additional cohorts are needed to replicate our findings, including those obtained with the PRS analysis. Moreover, our genetic correlation analysis relies on self-report information from the UK Biobank, where individuals (aged 40+) are probably older than the average age of individuals diagnosed with HIV-1. Consequently, behaviors relevant to HIV-1 acquisition that may be more common in a younger cohort (e.g. drug use), could have been absent (or not reported) in the UK Biobank. Where we do find genetic correlations with HIV-1 risk, we cannot currently infer cause and effect, but this is something which may be achievable in the future via a Mendelian randomization design, once larger GWAS are able to detect robust genome-wide significant predictors of HIV-1 acquisition. Finally, the cytokine panel we investigated in association with PRS for acquisition was limited to 35 proteins, and it is possible that other inflammatory markers which we did not assess may be more relevant to this trait.

To conclude, our results show that HIV-1 acquisition genetics impinges upon behavioral, cellular, and immune factors. By leveraging on modern population genetic methods, our work provides a novel framework to study HIV-1 acquisition as a complex phenotype, advancing our understanding of the underlying risk factors. Our results suggest that in addition to environmental risk factors, there is a polygenic component to HIV-1 acquisition that should be explored further in clinical studies. In addition, our work supports the investigation of future intervention strategies surrounding education and smoking behavior. In particular, there needs to be studies investigating whether smoking simply represents a proxy for unhealthy behaviors, or whether it actively influences biological processes linked to HIV-1 acquisition. Similarly, CCL17 and *EFCAB14* should also be investigated with respect to their potential role as biomarkers for HIV-1 acquisition and as drug targets. As demonstrated here, GWAS can be very informative even when analyzing moderately sized cohorts, but its true potential to unveil the host genetic mechanisms influencing HIV-1 acquisition will likely only be unlocked with the creation of collaborative initiatives and analyses of larger cohorts.

## Material and Methods

### GWAS summary statistics

We obtained summary statistics from the largest HIV-1 acquisition GWAS meta-analysis to-date, performed by McLaren and colleagues, which analyzed 6,334 HIV-1-positive individuals and 7,247 HIV-1-negative population controls of European ancestry, collected in Europe, North America, Australia and Africa^10^. The HIV-1-positive group consisted of individuals who acquired HIV-1 by sexual contact (homosexual and heterosexual, N  =  3,311), via the parenteral route (injection drug use and transfusion, N  =  1,046), or by unknown means (N = 2,086). The authors of this GWAS found no association of genotype with specific means of acquisition. We excluded variants not genotyped across all studies in the meta-analysis, those with a minor allele frequency <0.01, and those located within the major histocompatibility complex (chromosome 6, 26–34 Mb), due to its complex linkage disequilibrium structure.

### Heritability estimates and genetic correlations

We applied LD score regression in LD Hub v1.9.1^19^ to estimate the heritability of HIV-1 acquisition and to identify genetically associated traits, based on 516 heritable phenotypes assessed in the UK Biobank^39^. The UK Biobank phenotypically characterized and genotyped 488,377 participants aged between 40–69 years, enabling LD Hub to test for genetic correlations between HIV-1 acquistion and traits of interest (e.g. disease states, behaviors and socioeconomic traits)^40^. Results were plotted in R^41^ using the “EnhancedVolcano” library^42^. Estimation of SNP h^2^ and genetic correlations were also performed using SumHer-GC, from the LDAK package^20^, with the genomic control feature enabled, to control for potential hidden population structure in the data, even though the authors of the original GWAS did not identify genomic inflation (see McLaren and colleagues (2013)^10^). In the LDSC model, all SNPs are assumed to contribute equally to the phenotype, whereas in the LDAK model (which is expected to capture a larger proportion of heritability), a SNP with higher frequency is expected to contribute more towards heritability than one with lower frequency in the population, and a SNP in a region of low linkage disequilibrium contributes more than one in a region of high linkage disequilibrium.

### Gene-level and gene set enrichment analyses

We used the Functional Mapping and Annotation of Genome-Wide Association Studies (FUMA) to identify genes and cell types associated with HIV-1 acquisition genetics, as described elsewhere^17,18^. Briefly, gene-level statistics and weighted p-values were generated from GWAS summary statistics, adjusting gene-level associations for gene size, SNP density and LD. Input SNPs were mapped to 18,439 protein coding genes using a 10 kb upstream and downstream window, and a Bonferroni cut-off was applied to determine significance (P cut-off = 0.05/18,439 protein-coding genes = 2.71 × 10^−6^)^19^. Next, the gene-set enrichment analysis used two single-cell RNA-sequencing datasets available in FUMA to identify cell types enriched for genes associated with HIV-1 acquisition under the false discovery rate of 10% (q < 0.10). Specifically, to identify particular tissues or organs involved in HIV-1 acquisition, we tested the transcriptional profile of 75 murine cell types (TabulaMuris_droplet_all) for enrichment with HIV-1 acquisition susceptibility genes. This dataset is a comprehensive collection of well-curated single cell transcriptome data from *Mus musculus*, containing information from 100,605 cells from 20 organs and tissues^43^. To identify specific neural populations that could drive the behaviors observed in the genetic correlations, we tested the transcriptional profile of 565 neuronal cell types (DropViz_all_level2) for enrichment with HIV-1 acquisition susceptibility genes. This dataset contains the transcriptional signature of 690,000 cells from the mouse brain, which have been previously used to investigate the cellular mechanisms of behavior^44^.

### The South East London Community Health Study

We aimed to assess how HIV-1 acquisition risk might be moderated by an individual’s immune profile prior to infection, and so we tested how polygenic risk for HIV-1 acquisition correlated with the expression of 35 inflammatory markers. We studied HIV-1-negative population controls from the South East London Community Health Study (SELCoH)^45–47^, where HIV-1-negative status was determined based on self-report. For further details on the full SELCoH study, please see Hatch *et al*.^43^. SELCoH aimed to investigate mental and physical health in the general population in London, UK. The subsample analyzed in our study consisted of 406 individuals for which both inflammatory and genetic data were available. The SELCoH study received approval from King’s College London research ethics committee, reference PNM/12/13-152. Informed written consent was obtained from all participants at the time of sample collection. The 35 inflammatory markers represent adequately expressed cytokines from an initial panel of 42, which were originally assayed in relation to major depression risk, as described previously^48^. The mean age of our sample was 48.7 ± 15.1 (standard deviation), with a mean body mass index of 27.3 ± 5.5. The cohort is representative of the source population and consisted of 45.3% males; 20.9% current smokers and 40.4% ex-smokers; and 56.8% White British, 8.4% Black Caribbean, 10.9% Black African, 14.6% White Other, 6.2% Non-White Other, and 3.2% Mixed. Participants received detailed and repeated phenotypic assessments as part of three separate phases. The first phase aimed to assess common physical and mental disorders in South East London; the second, to examine the roles of historical social context and policy in shaping patterns of health inequalities; and the third, to collect biological specimens from a subset of participants, including blood for serum separation and DNA for genotyping. Serum and DNA were extracted and stored at −80 °C until use, as described previously^47,48^.

### Quantification and analysis of cytokines

Serum levels (pg/mL) of 35 blood-based markers were assessed in blood samples from the SELCoH cohort using multiplex ELISA-based technology provided by the Meso Scale Discovery Biomarker kits, as described previously^48^. For each marker, we adjusted for the effects of assay run, age, gender, body mass index (BMI), smoking (never, current, former) and ethnicity, by taking standardized residuals (z-scores). Z-scores were then used in downstream PRS analyses.

### DNA genotyping

DNA samples from the SELCoH cohort were sent to the Affymetrix Research Services Laboratory in Santa Clara, California, USA. Genotyping was assayed using the UK Biobank Axiom Array (r3) which comprises of 820,967 genetic markers (Affymetrix, California, United States). Genotype data was put through quality control measures as described previously, and used to construct polygenic risk scores^47^.

### Polygenic risk scores

Individualized polygenic risk scores (PRS) within the SELCoH sample were calculated using PRSice-2, a PRS quantification software^33,34^. This pipeline uses summary statistics from a base GWAS to generate individualized PRS in a target dataset. Briefly, the number of risk alleles in the base dataset are multiplied by SNPs’ effect sizes to generate individualized PRS in the target dataset. PRSice-2 clumps SNPs in the genotype files of the target dataset and removes those in high LD to avoid polygenic score inflation. For our initial screen, we constructed a PRS using all SNPs in the GWAS with P < 0.5. We tested whether this score predicted adjusted inflammatory marker levels in PRSice-2, covarying for seven population dimensions generated using principal component analysis applied to LD-pruned SNPs in PLINK 1.9^49^. Our relaxed p-value threshold (P_T_) was initially selected based on earlier studies which revealed that highly polygenic phenotypes in moderately powered GWAS are better captured using a relaxed cut-off ^50^. We subsequently performed sensitivity analyses for significant associations using a wide range of p-value thresholds, from p = 0.001 to p = 0.5, with 0.001 increments. This allowed us to determine the optimal P_T_ that explained the most variance. Furthermore, as part of a sensitivity test, once an optimal polygenic predictor was established, we tested its ability to predict inflammatory markers: (i) once the MHC region was excluded, (ii) when applied to individuals of European-only ancestry. This was performed to confirm that the result was not driven by confounding effects related to the complex structure of the MHC region, or to the genetic admixture in the SELCoH sample.

## Supplementary information

Supplemental Tables.

## Data Availability

Summary statistics were made available to us upon request to McLaren and colleagues^10^. Due to ethical restrictions, the SELCoH data is not publicly available. Details on the SELCoH sample and requests to access phenotype and inflammation data can be made at http://www.slam.nhs.uk/research/selcoh/selcoh-projects. Access to SELCoH genetic data requires local approval via the NIHR Bioresource (bioresource@kcl.ac.uk). Genetic correlations were performed using LD Hub and UK Biobank data, which are open access (http://ldsc.broadinstitute.org/ldhub/).
